# Principles for consistent value assessment and sustainable funding of orphan drugs in Europe

**DOI:** 10.1186/s13023-015-0269-y

**Published:** 2015-05-03

**Authors:** Laura Gutierrez, Julien Patris, Adam Hutchings, Warren Cowell

**Affiliations:** Celgene International Sàrl, Route de Perreux 1, Boudry, Switzerland; Celgene bvba/sarl Parc de l’Alliance, Boulevard de France 9, Braine L’Alleud, Belgium; Dolon Ltd, 17-185 Gray’s Inn Road, London, UK; Celgene Ltd, 1 Longwalk Road, Stockley Park, Uxbridge, London, UK

**Keywords:** Orphan medicinal products, Rare diseases, Value assessment

## Abstract

The European Orphan Medicinal Products (OMP) Regulation has successfully encouraged research to develop treatments for rare diseases resulting in the authorisation of new OMPs in Europe. While decisions on OMP designation and marketing authorisation are made at the European Union level, reimbursement decisions are made at the national level. OMP value and affordability are high priority issues for policymakers and decisions regarding their pricing and funding are highly complex. There is currently no European consensus on how OMP value should be assessed and inequalities of access to OMPs have previously been observed. Against this background, policy makers in many countries are considering reforms to improve access to OMPs. This paper proposes ten principles to be considered when undertaking such reforms, from the perspective of an OMP manufacturer. We recommend the continued prioritisation of rare diseases by policymakers, an increased alignment between payer and regulatory frameworks, pricing centred on OMP value, and mechanisms to ensure long-term financial sustainability allowing a continuous and virtuous development of OMPs. Our recommendations support the development of more consistent frameworks and encourage collaboration between all stakeholders, including research-based industry, payers, clinicians, and patients.

## Background

The growth in the number of approved orphan medicinal products (OMPs) in Europe since the introduction of legislation in 2000 has been a success for rare disease patients, the scientific and medical community, European and national policymakers, and the pharmaceutical industry. To date, one hundred and twelve (112) OMPs [1] have received marketing authorisation in Europe for the treatment of rare diseases associated with high unmet need and limited suitable therapeutic options [2,3]. Given the estimated 5000–8000 rare diseases that affect 6–8% of the European Union (EU) population (between 27 and 36 million people [4]), greater momentum in OMP development and approval will be necessary if the public health burden of rare diseases is to be addressed.

Whether this goal is reached will be determined in part by the outcome of an ongoing debate about the value of OMPs and the mechanisms by which European healthcare systems assess, reimburse and fund such treatments [5-9]. While the regulatory framework has been adapted to the specificities of OMPs by creating an expert Committee for Orphan Medicinal Products (COMP) within the European Medicines Agency (EMA), many national pricing and reimbursement systems have not been adapted to respond to the specific challenges of these types of drugs.

Regional and local inequalities of access to OMPs have previously been observed in Europe, where Member States have responsibility for health policy [10-13]. Such variations can be due to restrictions of reimbursement or funding, either because of a negative assessment by policymakers or because the drug has not been considered for reimbursement [10,11,14-17]. Negative reimbursement decisions can reflect problems in applying standard pricing and reimbursement processes to OMPs [18]. An awareness of this issue has led some European countries to adapt their processes to reflect the specificities of OMPs [6,12,19-22]. For example, in the United Kingdom (UK) the National Institute for Health and Care Excellence (NICE) recently created a separate programme for ultra-rare drugs that uses different assessment criteria to those applied to treatments for more common disorders [23,24].

Despite important initiatives to improve conformity of OMP assessment methods for reimbursement purposes at a European level, there is as yet no consensus on how OMP value should be evaluated [20]. This uncertainty has important consequences for healthcare policy makers, manufacturers and patients. Healthcare policymakers may lack confidence in the ability of their healthcare systems to ensure optimal patient access to OMPs while ensuring value for money and long-term financial sustainability. Manufacturers often face an unclear, inconsistent and changing array of systems and policies that create uncertainty about the long-term return from OMP development and provide little clarity of the data requirements to adequately ensure patient access [14].

Given the magnitude of the public health challenge in rare diseases it is important that pricing and reimbursement systems for OMPs are reformed to provide more consistent assessment of value and to be more closely aligned with regulatory frameworks for OMPs. This paper addresses this problem from the perspective of a biopharmaceutical manufacturer that has invested – and continues to invest – significantly in OMP research and development. It seeks to contribute to the debate on the OMP model by proposing a set of principles to improve the consistency, effectiveness and sustainability of OMP value assessment mechanisms in Europe, while maintaining flexibility and innovation in decision making between countries (Table 1). Ultimately, the ambition is to create a European OMP value framework that rewards innovation and ensures sustainability of the OMP model.

**Table 1:** **Overview of principles**

**Sections**	**Principles**
**Value Assessment**	1: National pricing and reimbursement processes should acknowledge the EMA’s assessment of therapeutic benefit
	2: National Authorities should incorporate rare disease expertise within their local assessment processes
	3: OMP assessment should consider all relevant elements of value
	4: Value assessment methods for OMPs should incorporate multiple criteria
	5: Value mechanisms should be flexible to accommodate evidential uncertainty at time of OMP approval
**Innovation and Price**	6: Adequate funding should be provided to ensure optimal patient access to OMPs and to incentivise research
	7: OMP reimbursement decisions should be determined by benchmarking value and price against treatments with similar characteristics
	8: If used, ICER thresholds should be modulated to reflect the specificities of rare diseases and OMPs
**Sustainability of OMP Model**	9: National authorities should develop adaptive and efficient processes to optimise use of real world data collected before and after value assessment
	10: Rational and evidence-based funding mechanisms should be developed to guarantee long-term sustainability

## Discussion

### Value assessment of OMPs

#### Principle 1: National pricing and reimbursement processes should acknowledge the COMP’s assessment of therapeutic benefit

The European OMP Regulation defines rare diseases as having a prevalence of no more than 5 in 10,000 people, and as diseases that are life-threatening or chronically debilitating [2,3]. Due to their inherent rarity and complexity, these diseases are often less well characterised or understood. There is often limited information on rare disease pathophysiology, mechanisms or genesis, a lack of relevant animal models and problems with accurate diagnosis [25]. Information on the disease process, epidemiology, prognosis, and burden is also often incomplete. Current standard of care treatments may be poorly established with little evidence of effectiveness [26]. As well as a lack of information about the disease as currently managed, evidence for new treatments can be difficult to interpret [27].

There is therefore a need for specific expert involvement in the benefit assessment of OMPs, a point recognised in the conception of OMP Regulation [28]:

*“Gathering expertise at European level is therefore paramount in order to ensure equal access to accurate information, appropriate and timely diagnosis and high quality care for rare disease patients.”*

In establishing the COMP within the EMA, the OMP Regulation provided for such expert involvement in regulatory decision-making. In addition, the OMP Regulation set out specific criteria for the assessment of a medicinal product that applies for an OMP designation and authorisation [2,29].

In the criteria to obtain the designation of orphan drug (Article 3 of Regulation (EC) 141/2000) it is clearly stated that a medicinal product shall be designated as an OMP where

1. “*there exists no satisfactory method of diagnosis, prevention or treatment of the condition in question that has been authorized in the European Union or”,*
2. *“if such method exists, that the medicinal product will be of significant benefit to those affected by that condition*”.

The European regulatory framework for OMPs therefore went beyond the safety, efficacy and quality requirements for other drugs by specifying that for OMPs, manufacturers also need to demonstrate in certain cases that the product provides significant benefit for the targeted patient population, defined as ‘a clinically relevant advantage or a major contribution to patient care’ [30].

Given the rare disease expertise that exists within the COMP, it is important for national reimbursement mechanisms to be consistent with the scientific assessment performed under the European regulatory decision making process [31,32]. National, regional and local pricing and reimbursement authorities remain competent with regards to the funding of OMPs, but their assessment should acknowledge the existence of a clinical advantage, when such benefit has been established through OMP designation [18].

This principle is incorporated into the pricing and reimbursement rules in Germany. The “innovativeness” and the added medical benefit for OMPs is assumed by law (Sec. 35a, subs. 1 clause 10 Social Code Book Volume V, Sozialgesetzbuch Band V – SGB V), representing a deferment by the Federal Joint Committee to the COMP assessment regarding OMP benefit [33]. This deferment is limited to OMPs with a yearly turnover of less than EUR 50 million.

#### Principle 2: National authorities should incorporate rare disease expertise within their local assessment processes

As with the regulatory process, national policy makers should ensure local disease specific expertise is incorporated into the value assessment process [16]. In the common absence of robust observational data, expert insight – both from health care professionals (HCP) and patients – is very important [14]. Thus when assessing value in rare diseases, there should be careful consideration of qualitative evidence alongside the quantitative. A full understanding of these often complex diseases can only be achieved through testimony from HCPs, patients, carers and family members.

The importance of involving patients in the development of medicines has become widely recognised and increasingly accepted. Patient experts provide input into the design and endpoints of clinical trials, have a voice in regulatory decisions and are frequently important stakeholders in health technology assessment (HTA) processes [34,35]. First hand testimony on the patient experience can help to redress the absence of published observational data in rare diseases and help policymakers understand the relevance and context of unfamiliar clinical endpoints [18,36,37].

Patient involvement is particularly important in rare diseases because the difficulty patients face in obtaining appropriate care mean that they often have no choice but to become experts in their own condition. Equally, physicians managing patients with rare diseases might not always see a sufficient volume of patients to be able to correctly characterise the full range of symptoms and disease manifestations across different patient segments [16].

In practice, this implies a need for more systematic and meaningful involvement of patients and HCPs into value assessment processes for OMPs. At a national level, rare disease expert involvement is important because representatives on standing committees within assessment agencies may never have encountered a particular rare disease, and may have no specific knowledge of the patient experience [16].

Representation from experts with knowledge of treatment patterns for a rare disease at a national and international level is key. Input from multiple disease experts and centres may be required [31]. Clinical experts are likely to be chosen from reference centres for the disease in question within the country in which the value assessment is taking place. For diseases where there is no available national expertise, a European or international expert could be consulted [37]. Clinical expertise should be sought not just from physicians but also nurse experts who can often provide additional context on the full care pathway for patients, beyond pharmaceutical management.

The involvement of such experts will provide national authorities with a better understanding of the disease context, thus reducing the risk of an OMP being excluded from reimbursement without careful consideration of the specificities of the rare disease and their impact on its evaluation. In particular, such experts can provide national assessment bodies with information that might otherwise be unavailable on issues such as disease morbidity, patient disability and prognosis [16].

#### Principle 3: OMP assessment should consider all relevant elements of value

There are some key specificities of rare diseases and OMPs that are essential to their value, but are not consistently considered in national value assessments, such as disease severity, the lack of a suitable alternative treatment (unmet need) and rarity [6,16,38].

Traditional measures of health outcome such as Quality- and Disability-Adjusted Life Years (QALY/DALY) are not always sensitive to the disease severity of the patient population [6,27]. Proportional improvements in health outcome generate smaller QALY gains in severe patient populations than in healthier ones. This particularly affects OMPs as by definition they target life-threatening or chronically debilitating diseases [6,39]. Patients often have a very poor prognosis and quality of life compared to the general population. In most cases, even with the introduction of a new therapeutic option with proven benefits, the health status of these patients will remain relatively modest compared to the general population. In this context traditional health outcomes analysis might conclude that the clinical benefit is modest [6,39]. However, due to the severity of these diseases, for these particular patients, the clinical benefit is important.

The absence of effective or well-documented standard alternative treatments also complicates the assessment of OMPs. Frequently, there is disagreement about which treatment to use as the comparator within an OMP assessment [6,39]. The comparators used for price benchmarking of OMPs are often old, generic and without proven efficacy (and sometimes used off-license). Even if the standard of care is recognised as ineffective, it will still be a price benchmark in many pricing and reimbursement systems [6]. In addition, in cases where the current standard of care treatments may be poorly established, there is often a lack of information on the additional healthcare costs associated with poorly controlled disease. As a result, the incremental cost of OMPs is overstated, leading to complex discussions between the manufacturer and authorities. Thus, the lack of a suitable alternative treatment – the main driver for OMP legislation – can negatively affect OMP value assessment by authorities. To counter this effect, unmet medical need should be recognised as a significant component of the value of a new drug [6,16,38].

Differences in disease prevalence also have implications for the level of disease understanding, the quality of evidence of treatment benefit and the price of the drug [40]. While rarity is not necessarily considered an important element of value *per se*, it ought to be considered during assessment due to its effect on these other factors.

#### Principle 4: Value assessment methods for OMPs should incorporate multiple criteria

Multi-criteria assessment methods offer an opportunity to fully incorporate all relevant elements of OMP value into a funding decision in a structured, transparent and consistent manner [41]. This is in contrast to HTA methods based on cost effectiveness analysis (CEA) which usually include a narrower range of value elements and provides less flexibility in the decision-making process [6].

A number of authors have proposed multi-criteria assessment frameworks for OMPs, arguing that they allow for the explicit consideration of rare disease specificities [7,42,43]. For example, the European Commission Working Group on Mechanism of Coordinated Access to Orphan Medicinal Products proposed a Transparent Value Framework for OMPs that utilised a multi-criteria approach [44]. Similar multi-criteria methods are used within the English and French healthcare systems when making funding decisions on rare disease treatments [16,45].

The advantage of multi-criteria assessment for OMPs lies in providing a structured framework that enables the systematic and transparent incorporation of value elements important to the decision situation [46,47]. They can formally incorporate OMP specific elements (such as rarity, severity, unmet need), and assess the degree of added-value they represent for the health care system [42,48]. In addition, multi-criteria frameworks also provide the flexibility to allow for the incorporation of more subjective factors, such as ethical issues relating to rare disease patient access to treatment, the rule of rescue, equity of opportunity for patients to benefit, and the patient’s perspective.

#### Principle 5: Value mechanisms should be flexible to accommodate evidential uncertainty at time of OMP approval

The existence of evidential uncertainty does not mean that a product lacks value or is insufficiently researched. Clinical uncertainty affects nearly all innovative medicines at the time of approval, regardless of patient population sizes. Quantitative uncertainty, however, is greater in rare diseases because of smaller patient populations [14].

The willingness to accept uncertainty around OMP value parameters should be determined relative to the specificities of each disease: the prevalence and heterogeneity of the patient population, the level of knowledge of the disease, its natural history, the availability of established surrogate endpoints and the efficacy of comparator products [15,31].

Value assessment mechanisms that allow for both qualitative and quantitative assessment of clinical benefit are more likely to be sensitive to the context of data generation than purely quantitative approaches, such as CEA [49]. Nevertheless, statistical uncertainty is an inherent problem in rare diseases, regardless of assessment model, and efforts should be made to ensure it is not a systematic impediment to OMP access [50]. Mechanisms to manage this uncertainty and to improve understanding of clinical benefit are discussed in Principle 9.

### Innovation and price

#### Principle 6: Adequate funding should be provided to ensure optimal patient access to OMPs and to incentivise research

In the 30 years preceding the introduction of the European OMP Regulation pharmaceutical innovation was focused on more common diseases, reflecting scientific research priorities, aggregate patient need and commercial viability [51]. The resulting improvement in care levels led to a greater differential in health status and treatment options between individuals with prevalent diseases and those with rare diseases. The OMP Regulation was an explicit incentive to encourage pharmaceutical companies to prioritise investment in rare diseases with unmet need and to redress the imbalance in opportunity for health gain [26,49,52]. However, although this goal is explicit in OMP Regulation, it is not always a specific objective of national health policy programmes [2,53] nor pricing and reimbursement frameworks.

OMP funding is contentious, primarily because of the sometimes high per-patient cost of OMPs (although some OMPs have similar prices to non-OMP drugs) [54]. It is generally accepted that the small patient numbers in rare diseases mean that the cost per patient of OMPs is likely to be higher than treatments for more prevalent conditions [55]. This is inevitable if manufacturers are to recoup research and development expenditure and be incentivised to develop treatments for rare diseases [54-57].

Payers and policy makers within each Member States should acknowledge the need to incentivise OMP development through pricing and reimbursement systems, as OMP development is impossible if prices are constrained to those of drugs for common diseases with bigger patient populations [6]. Companies invest in OMPs because, if successful in developing effective treatments, they expect positive returns on investment [54]. By prioritising rare disease research, policymakers endorse positions established in the late 1990s that encouraged pharmaceutical companies to shift Research and Development (R&D) emphasis away from “me-too” drugs in prevalent primary care diseases towards innovative treatments for severe diseases in which treatments were unavailable [6]. Without some allowance for higher OMP prices, it is unlikely that the recent growth in research investment in this field will continue, and the considerable need in rare diseases will remain unmet [49]. Similarly, given the small numbers of patients affected, and the great challenges inherent in attempting to predict patient response, decisions to restrict reimbursement to sub-groups within the licensed indication should be a last resort.

When innovation is successful, access to this innovation will inevitably require, in the short-term, additional investment from healthcare. The recent growth in orphan drug expenditure is a natural consequence of the recent innovation in disease areas that were hitherto untreatable [14,54]. However, the growth of OMP expenditures is expected to be manageable over time and should not undermine the sustainability of the healthcare system. Currently, OMP expenditure is estimated to account for 1%–4% of all pharmaceutical expenditure [54] and is expected to grow to approximately 5% by 2020 remaining stable overtime [58]. The end of patent protection and marketing exclusivity will allow the introduction of generics and price reduction, this will create financial room for the introduction of new orphan drugs [58]. This principle, however, does not exempt pharmaceutical companies from their obligation to justify their prices based on a sound value assessment.

#### Principle 7: OMP reimbursement decisions should be determined by benchmarking value and price against treatments with similar characteristics

Working within a flexible value framework, OMP reimbursement should be determined according to the added value derived from treatment as assessed through an appropriate process that captures the most relevant rare disease value elements (see Principle 3). It is clearly understood that there is, and will be, variability in the value of OMPs [36], and pricing and reimbursement frameworks should reflect this. Added value must be established in a way that allows for comparisons with other OMPs with similar characteristics, including rarity, disease severity or drug development complexity. By establishing added value relative to a cohort of similar OMPs, the reimbursement can accordingly be determined against the spectrum of prices for that type of OMP.

Funding status would be established relative to the price of other similar treatments and conditions rather than to the price of existing treatments for the same disease (as occurs in common disorders). Because of the nature of OMPs which, by definition, are for diseases in which no adequate treatments are available, the prices of existing treatments are insufficient to incentivise OMP development [57]. Instead, OMP prices should be anchored within the current price range of OMPs for diseases with a similar prevalence, with the specific price determined by the added value relative to those other treatments.

#### Principle 8: If used, ICER thresholds should be modulated to reflect the specificities of rare diseases and OMPs

There has been considerable debate in OMP policy literature about the appropriateness of using CEA as a decision-making tool in rare diseases [6,38,59-61]. Uncertainties around rare disease clinical parameters, combined with unknown economic variables, can render such a broad range of incremental cost-effectiveness ratios (ICERs) that they are often meaningless [55,62]. While many authors have suggested that CEA should play a limited role in OMP assessment, others have argued that it is feasible, provided adjustments are made to reflect OMP specificities [6,7,43,63].

Experience to date suggests that the use of a multi-criteria framework might be more suitable than an OMP-adapted CEA. Countries such as France and Italy, where economic analysis is used adjunctively, rather than as the driver of decisions, have been seen to have relatively high patient access to OMPs [11,15].

If a CEA framework is used, ICER thresholds should be modulated accordingly. Changes to ICER thresholds should adjust for value criteria that are not routinely captured within a CEA framework, such as the disease severity, unmet need (availability of alternatives) and disease prevalence. Weighting should be explicit and consider established public policy choices and societal preferences to encourage OMP development [7,43,63,64].

Higher ICER thresholds have been seen to have some success for certain types of OMPs – particularly treatments for rare oncological diseases – where trial designs and endpoints have been more conducive to CEA assessment. For example, in England end-of-life guidance allowed a higher ICER of up to £50,000 per QALY for patients with a particularly poor prognosis [65]. Six of nine oncological OMPs that have been appraised under this exemption have received a full or partial recommendation [66]. However, for many non-oncological OMPs, this approach has not improved access.

### Sustainability of OMP model

#### Principle 9: National authorities should develop adaptive and efficient processes to optimise use of real world data collected before and after value assessment

Pricing and reimbursement assessments of OMPs are complicated by uncertainty relating to the clinical profile and evidence base of a new treatment. Standard pharmaceutical value assessment processes place great emphasis on the quantity and quality of clinical data, as measured by statistical measures of variance and the familiarity of endpoint metrics [6,27]. The nature of rare disease research makes it extremely hard to generate sufficient data to meet the evidence levels expected of treatments for common disorders within HTA processes [18,31,37,67].

Early access programmes offer opportunities for policymakers to incorporate real-world, locally-derived data into the value assessment process while facilitating patient access to new drugs for diseases with great unmet need. Such programmes have been effective in France, Italy and the Netherlands [20,68]. For example, the French Autorisations Temporaires d’Utilisation (ATU) scheme provides early access to new promising treatments where a genuine public health need exists [69,70]. To qualify, there must be no available satisfactory alternative treatment and the new treatment must show expected clinical benefit, and an acceptable benefit/risk ratio; these criteria are often relevant for OMPs [71]. Data collection is mandatory within the ATU scheme, which can then be referenced by the Transparency Committee during value assessment. In total, 72% of the 64 authorised OMPs were available through the ATU scheme [71], ensuring high levels of OMP patient access in France.

Patient registries and post-authorisation safety studies offer opportunities to capture patient-level data on clinical and economic outcomes and are increasingly standard requirements as part of local registration and policymaker assessment [5,19,32,37,60,72]. Registries for OMP indications should be flexible enough to collect sufficient data and account for evolutions in patient populations and treatments over time. It has been recommended that registries be standardised at the EU level with coordination of evidence requirements between the EU and member states [72], for instance, through greater collaboration with the COMP [73].

In cases where clinical uncertainty is deemed too large at the time of reimbursement, policymakers may link reimbursement to subsequent real world outcomes [5,18,31,32,39,43,55,67,74]. Conditional reimbursement programmes, whereby OMPs are reimbursed if they prove effective in real world settings, can be combined with registries to allow on-going monitoring of their use, efficacy and value. These should form part of post-marketing surveillance programmes that will also monitor OMP uptake [5,18,31,32,39,55,67,74].

#### Principle 10: Rational and evidence-based funding mechanisms should be developed to guarantee long-term sustainability

Financial uncertainty around OMP expenditure is a general concern because of potential budget impact variability. Authorities are rarely able to accurately predict the uptake rate of new medicines, a situation exacerbated in rare diseases by the relative paucity of epidemiological data on the number of patients for whom the medicine might be appropriate [67]. Where OMPs have high per-patient prices, small changes in patient numbers can result in significant budget impact changes compared with initial forecasts. However, while the variation in budget impact for a single drug might be quite high, variation in aggregate OMP expenditure is likely to be much lower [54] and the emphasis on reducing financial uncertainty at a drug level may therefore, be less important than initially assumed.

To manage a specific risk of financial uncertainty that could endanger the sustainable introduction of new OMPs over time, specific financial mechanisms may be established. These may include price/volume agreements, budget caps, and ‘payback’ mechanisms linked to budget impact forecasts agreed with policymakers at reimbursement [6,19,55,59-61,68,72,74]. But it should be the policymaker’s responsibility to demonstrate that there is sufficient financial uncertainty around a new product to unbalance sustainability of OMP funding. Without this requirement, there is a risk that such mechanisms will be used routinely to reduce cost and undermine OMP value. Authorities should be careful not to label situations where the price is considered excessive as ‘uncertain’. True uncertainty regarding budget impact is principally related to patient life expectancy and the number of patients likely to be treated. The management of the growth of OMP expenditures should be primarily done through solid and consistent decisions regarding prices and reimbursement of new products.

## Summary

The OMP Regulation can be considered an example of successful health policy making. Recognition of the considerable unmet need for rare disease patients led to policy makers explicitly prioritising the development of treatments for these conditions. As a result the pharmaceutical industry quickly responded to these incentives. The Orphan Medicinal Product Regulation has thus contributed to boosting research, development and marketing of OMPs in Europe after decades in which no new treatments were approved.

However, OMP access is still variable in Europe, reflecting a lack of consensus between countries on how to assess the value of OMPs and what constitutes a fair price [18]. The discussions on this topic reflect a broader debate concerning on the one hand, the sustainability of funding, while on the other, acknowledging the need for continuous research into rare diseases. Addressing these challenges is a shared concern. Thus to contribute to the debate this paper puts forward a number of practical recommendations from the perspective of a pharmaceutical manufacturer to help provide a more consistent value assessment framework for orphan drugs that rewards innovation and ensures the sustainability of the OMP model.

Value assessment frameworks should be adapted to OMP specificities. Practically, this means ensuring the involvement of rare disease experts in national and local reimbursement decision making processes as well as the acknowledgement by national and local authorities of the expertise within the EMA COMP and consideration for its assessment of added therapeutic benefit. Pricing and reimbursement systems at national levels do not unanimously address specific OMP characteristics when considering their value, thus affecting patient access to OMPs. Value assessment frameworks should be flexible enough to reflect the specificities of rare diseases, such as the disease burden and lack of therapeutic alternatives [6,16,38]. There is a growing consensus that multi-criteria frameworks are particularly well-suited to capturing these specificities in rare diseases [42,43,64]. Patient access to OMPs can be optimised by using economic analysis adjunctively, rather than relying on incremental cost-effectiveness as the main driver of decisions.

Policymakers can account for higher clinical uncertainty in OMP compared to other drug valuations through adaptive reimbursement processes. The magnitude of the unmet need in rare diseases creates an imperative for OMPs to be quickly accessible to patients. Emphasis should be on demonstrating clinical benefit and safety, while generating further value data post-authorisation, for example, in the form of European registries.

Finally, if the burden of rare disease is to be reduced, the prioritisation of rare diseases by policy makers through the OMP Regulation needs to be matched by the explicit prioritisation of funding for OMPs. Prices for rare disease treatments should be benchmarked against treatments with similar characteristics according to the degree of added value provided. Such price levels have been proven to be sufficient to incentivise a significant shift in R&D investment. Such incentives should be balanced against the needs for budget sustainability, which should be achieved through financial risk-management mechanisms. However, it is important that funding considerations should not be conflated with the assessment of clinical benefit – funding matters should be considered separately and subsequently to the assessment of added value.

In summary, three elements are necessary to balance the needs for financial sustainability against the need for continuous development of OMPs: the continued prioritisation of rare diseases by EU and national policymakers, an appropriate pricing framework centred on OMP value, and mechanisms to ensure long-term financial sustainability of OMPs.

## Abbreviations

ATU: Autorisations temporaires d’utilisation
CEA: Cost-effectiveness analysis
CEESP: Commission d’évaluation économique et de santé publique
COMP: Committee for orphan medicinal products
EMA: European Medicines Agency
EU: European Union
HTA: Health technology assessment
ICER: Incremental cost-effectiveness ratio
NICE: National Institute for Health and Care Excellence
OMP: Orphan medicinal product
R&D: Research and development
UK: United Kingdom
